# Can virtual non-contrast imaging replace true non-contrast imaging in multiphase scanning of the neck region?

**DOI:** 10.1177/20584601231205159

**Published:** 2023-09-25

**Authors:** Zaid Al-Difaie, Max HMC Scheepers, Nicole D Bouvy, Sanne Engelen, Bas Havekes, Alida A Postma

**Affiliations:** 1 GROW School for Oncology and Developmental Biology, 82246 Maastricht University, Maastricht, The Netherlands; 2 Department of Surgery, 199236 Maastricht University Medical Center, Maastricht, The Netherlands; 3 Division of Endocrinology and Metabolic Disease, Department of Internal Medicine, 199236 Maastricht University Medical Center, Maastricht, The Netherlands; 4 Department of Radiology and Nuclear Medicine, School for Mental Health and Neuroscience, 199236 Neuroradiology, Maastricht University Medical Center, Maastricht, The Netherlands

**Keywords:** diagnostic, computed tomography, head and neck, dual-energy CT, virtual non-contrast imaging

## Abstract

**Background:**

Dual-energy computed tomography (DECT) is an advanced imaging method that enables reconstruction of virtual non-contrast (VNC) images from a contrast-enhanced acquisition. This has the potential to reduce radiation exposure by eliminating the need for a true non-contrast (TNC) phase.

**Purpose:**

The purpose is to evaluate the feasibility of VNC images in the neck region.

**Materials and methods:**

A total of 100 patients underwent a DECT scan as part of diagnostic workup of primary hyperparathyroidism. VNC images were reconstructed from 30 s (arterial) and 50 s (venous) post-contrast scans. Regions of interest (ROIs) were placed in thyroid tissue, lymph node, carotid artery, jugular vein, fat, and sternocleidomastoid muscle. Mean densities of all anatomical structures were compared between VNC and TNC images.

**Results:**

For all anatomical structures except the thyroid gland, the difference in mean density between TNC and VNC images was less than 15 HU. The mean difference in density between TNC and VNC images of the thyroid was 53.2 HU (95% CI 46.8; 59.6, *p* = <0.001).

**Conclusion:**

This study demonstrated an acceptable agreement in density between true non-contrast and virtual non-contrast images for most anatomical structures in the neck region. Therefore, VNC images may have the potential to replace TNC images in the neck. However, due to significant differences in CT density of thyroid tissue, true non-contrast imaging cannot be directly substituted by virtual non-contrast imaging when examining the thyroid and its surrounding tissue.

## Introduction

Dynamic multiphase contrast-enhanced CT (4D-CT) is an accurate diagnostic technique that uses multiple phases of contrast-enhanced CT scanning. This technique can be employed in the neck region, such as for detecting parathyroid adenomas.^1^ However, a significant drawback of 4D-CT is the relatively high radiation exposure that results from the multiple scanning phases.^2^ This has led to concerns about the potential for an increased risk of thyroid cancer. Therefore, it has been argued by some authors to use 4D-CT judiciously, especially in younger patients.^2^

In the last decade, dual-energy computed tomography (DECT) has gained increasing interest in diagnosing head and neck conditions, offering improved tissue characterization and differentiation through the use of two different photon spectra.^3–8^ DECT acquisition can be achieved by several different techniques including two separate X-ray tubes (dual-source), single source scanners with rapid KV switching systems, dual-layer spectral CT, and photon counting.^9^

One of the advantages of DECT-scanning is the ability to reconstruct virtual non-contrast (VNC) images from a contrast-enhanced acquisition. This may reduce radiation exposure by eliminating the need for a true non-contrast (TNC) phase.^10^ This reduction in radiation exposure using VNC images may be particularly beneficial in the neck region since the effective dose in the neck is influenced by the relatively high sensitivity to radiation of the thyroid and salivary glands.^11,12^

To achieve radiation reduction by eliminating the true non-contrast phase, VNC images should replace TNC images. In order to substitute TNC images, an acceptable agreement in density is required between TNC and VNC images. Typically, a difference in density of 10 to 15 Hounsfield units (HUs) is accepted as the maximum accepted difference in density for substituting TNC with VNC images.^13,14^ At present, there is no consensus on the ability of VNC images to substitute TNC images in head and neck imaging.

The feasibility of VNC images has been extensively investigated for various indications, with several studies demonstrating its ability to reduce radiation exposure without reducing diagnostic accuracy.^10,15–18^ However, there is a paucity of studies specifically investigating the feasibility of using VNC images in the neck region. We hypothesize that VNC images cannot be used as a substitution for TNC images in head and neck imaging, as it is expected that the intrinsic iodine of the thyroid is subtracted during the reconstruction of VNC images. Therefore, the aim of this study was to determine the feasibility of using VNC images in the neck region.

## Material and methods

### Patient selection

This was a retrospective study that included all consecutive patients who underwent 4D-DECT imaging as part of standard diagnostic workup of primary hyperparathyroidism between June 2016 and November 2022. Clinical and demographic data were retrieved from electronic patient records and images were retrieved from the PACS system. The study protocol was approved by the Medical Ethics Commission (17-4-077.2). Informed consent was waived.

### CT technique

4D-CT was performed on a third-generation dual source CT scanner (Somatom Definition Force, Siemens, Erlangen, Germany). The imaging protocol consisted of three phases: (1) a true non-contrast scan (TNC) (SECT *n* = 43 or DECT *n* = 57), (2) a DECT post-contrast scan at 30 s (arterial), and (3) a DECT 50-s post-contrast scan (venous). The post-contrast scans were conducted after administration of 70 mL Ultravist 300 (Bayer Schering) with a flow rate of 3 mL/s, followed by 30 mL saline flush at the same flow rate. Scan parameters were as follows: SECT scans were scanned in a care-kV energy mode. DECT at tube voltages 80/150 kVp and 35/23, Quality Reference mAs, CTDIVol 5.6 mGy, rotation time 500 ms, pitch 0.7, 1 mm slice thickness, FOV 270 mm, matrix 512 × 512, and collimation 0.6.

A soft tissue kernel was applied for all series. Mixed images were reconstructed with a weighted average between the two tube sources of 0.5 with a soft tissue kernel. DECT data were reconstructed with a quantification kernel (Q30) for post processing.

VNC reconstructions were created by applying a three-material decomposition algorithm “Brain Hemorrhage” in a dedicated software package (Syngovia, version VB60, Siemens Healthcare) from arterial and venous phases.

### Image analysis

Scans were reviewed on a Syngovia workstation (for DECT measurements) and a PACS viewing station (Sectra, IDS7 version 24.2, Linkoping, Sweden). A circular ROI with a maximum diameter of 20mm^2^ was placed and densities were measured (mean HU and SD) in thyroid tissue (T), lymph node (LN), carotid artery (CA), jugular vein (JV), fatty tissue (FT), and sternocleidomastoid muscle (SCM) by a board-certified neuroradiologist with more than 15 years of experience. Care was taken for all ROIs to avoid confounding factors, such as thyroid nodules, blood vessels, and streak artefacts. ROIs were placed in each abovementioned anatomical structure in all phases (0, 30, and 50 s).

The contrast-to-noise ratio (CNR) was calculated for every anatomical structure using the following formula: (ROItissue – ROIscm)/SDfat, where ROItissue is the mean density of the tissue of interest (HU) and ROIscm is the mean density of the sternocleidomastoid muscle tissue (HU), and SD is the mean standard deviation of the density of subcutaneous fat.^19^ The signal-to-noise ratio (SNR) was calculated for every anatomical structure using the following formula: SNR=|ROItissue|/SDfat, where ROItissue is the mean density of the tissue of interest (HU) and SDfat is the mean standard deviation of the density of subcutaneous fat.^20^

### Statistical analysis

Descriptive statistics were used for baseline patient characteristics. The mean and standard deviation were used for continuous variables, and count and percentage for categorical variables. Mean densities of the thyroid, LN, CA, JV, FT, and SCM were compared between TNC and VNC scans. Furthermore, mean density between VNC scans constructed from 30 s post-contrast scans and 50 s post-contrast scans were also compared. Differences in density between TNC and VNC scans were tested using the paired-samples t-test.

The agreement between TNC and VNC images was visualized using Bland–Altman plots, which plotted the differences in density between TNC and VNC images against the mean density of the TNC and VNC images. The limit of agreement was determined as the mean difference ±15 HU.

The Wilcoxon signed-rank test was used to compare CNR and SNR between VNC and TNC images. All statistical tests were two-tailed and results with a *p*-value of less than or equal to 0.05 were considered statistically significant.

## Results

One hundred patients undergoing DECT scanning of the head and neck region for primary hyperparathyroidism were included; 23 patients were males and 77 patients were females. The mean age was 63.5 ± 10.7 years. Patient demographics are summarized in Table 1.

**Table 1.** Baseline characteristics (demographics).

Characteristic	*N*	Mean	SD	Min	Max
Age	100	63.5	10.7	30	80
TSH baseline	79	2.5	3.7	0.5	33.4
Sex (% female)	100	77%^a^			

SD: standard deviation; TSH: thyroid stimulating hormone.

^a^Percentage of females in total study population.

Mean densities of all anatomical structures and their differences on TNC and VNC images are presented in Table 2 and Supplemental Table 3. For all anatomical structures except thyroid tissue, the difference in mean density between TNC and VNC images reconstructed from 30 to 50 s was less than 15 HU (Table 2 and Supplemental Table 3); the difference between TNC and VNC images was less than 10 HU for carotid artery (CAR), jugular vein (JUG), and sternocleidomastoid muscle (SCM) (Table 2 and Supplemental Table 3).

**Table 2.** Differences in attenuation between native and virtual native scans (30s) for different tissue.

Tissue	TNC^a^	VNC (30s)^a^	Difference (95% CI)	*p*-value
Thyroid gland	101.1 (25.1)	47.8 (16.2)	53.4 (47.0; 59.8)	<0.001
SNR thyroid	9.2 (3.8)	4.4 (2.2)	—	—
CNR thyroid	3.6 (2.6)	−0.5 (1.7)	—	—
Lymph node	51.8 (11.2)	37.7 (8.4)	14.1 (11.5; 16.6)	<0.001
Carotid artery	50.7 (8.9)	43.4 (7.3)	7.3 (5.1; 9.5)	<0.001
Jugular vein	49.3 (6.9)	42.8 (6.6)	6.5 (5.0; 8.0)	<0.001
Sternocleidomastoid muscle	60.9 (5.4)	52.8 (6.0)	8.0 (6.6; 9.6)	<0.001
Fat	−112.7 (17.7)	−100.0 (11.1)	−12.7 (−16.0; −9.4)	<0.001
SNR thyroid	9.2 (3.8)	4.4 (2.2)	4.8 (4.1; 5.5)	<0.001
CNR thyroid	3.6 (2.6)	−0.5 (1.7)	4.1 (3.4; 4.8)	<0.001
SNR lymph node	4.8 (2.1)	3.4 (1.3)	1.3 (1.1; 1.6)	<0.001
CNR lymph node	−0.8 (1.2)	−1.4 (1.2)	0.6 (0.4; 0.9)	<0.001
SNR carotid artery	4.7 (1.9)	4.0 (1.7)	0.7 (0.4; 0.9)	<0.001
CNR carotid artery	−0.9 (1.1)	−0.9 (0.6)	−0.1 (−0.3; 0.2)	0.6
SNR jugular vein	4.6 (1.8)	3.9 (1.5)	0.6 (0.5; 0.8)	<0.001
CNR jugular vein	−1.0 (0.8)	−0.9 (0.8)	−1.0 (−0.3; 0.1)	0.08
SNR sternocleidomastoid muscle	5.6 (2.0)	4.9 (1.9)	0.7 (0.6; 0.9)	<0.001
SNR fat	−10.6 (4.4)	−9.3 (3.6)	−1.2 (−1.5; −0.9)	<0.001
CNR fat	−16.2 (6.3)	−14.2 (5.3)	−2 (−2.4; −1.6)	<0.001

CI: confidence interval; CNR: contrast-to-noise ratio; SD: standard deviation; SNR: signal-to-noise ratio; TNC: true non-contrast; VNC: virtual non-contrast.

^a^Data represent attenuation expressed in mean Hounsfield units with standard deviation between brackets.

**Figure 1.**
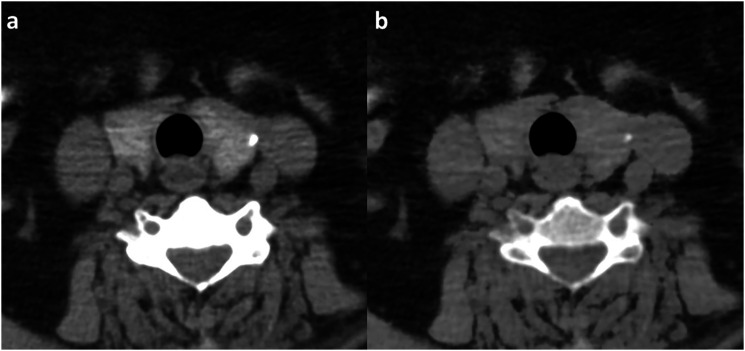
True non-contrast image (TNC) (a) and virtual non-contrast image (VNC) (b) detail of thyroid region of the neck of one patient. Window width and window level are comparable. Some streak artefacts are shown due to the location between the shoulders. The density of the thyroid differs between the TNC (120 HU) and VNC (63 HU). A small calcification can be seen in the left hemi-thyroid.

The mean difference in thyroid density between TNC and VNC images was 53.4 HU (95% CI 46.8 HU; 59.6 HU) at 30 s and 51.7 HU (95% CI 45.6 HU; 57.5 HU) at 50 s. An example of DECT scans of the thyroid region, revealing a significant reduction of thyroid density on a VNC image compared to the TNC image are provided in Figure 1.

There was no significant difference in mean density for all anatomical structures between VNC reconstructed from 30 to 50 s (Supplemental Table 4).

**Figure 2.**
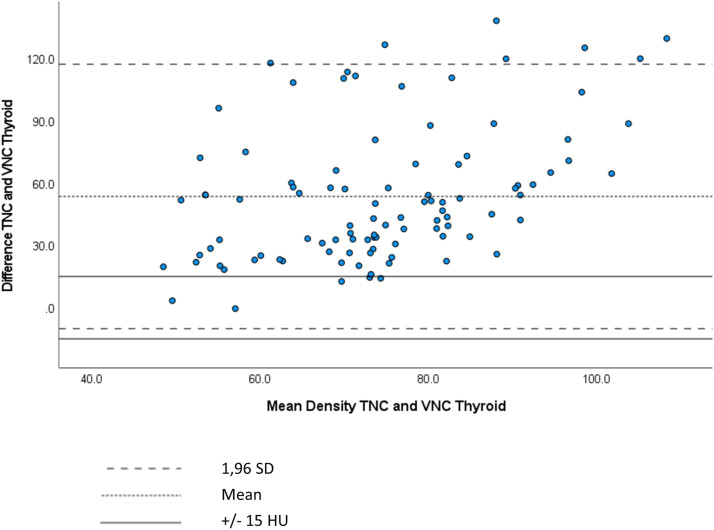
Bland–Altman plot showing differences in density between TNC and VNC images reconstructed from 30 s against the mean density of the TNC and VNC images of thyroid gland.

**Figure 3.**
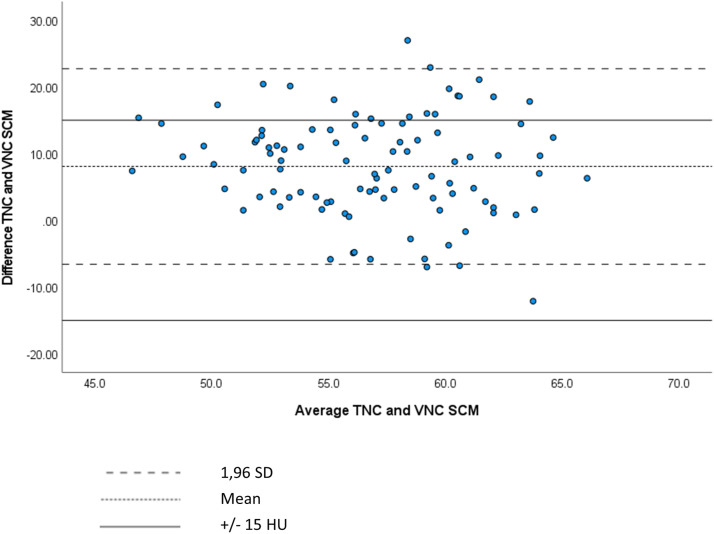
Bland–Altman plot showing differences in density between TNC and VNC images reconstructed from 30 s against the mean density of the TNC and VNC images of sternocleidomastoid muscle.

The agreement between TNC and VNC images was visualized using Bland–Altman plots, which plotted the differences in density between TNC and VNC images against the mean density of the TNC and VNC images. Bland–Altman plots revealed that 95% of differences fell between two lines of limits of agreement for each anatomical structure, except for thyroid tissue. A Bland–Altman plot [95% limit of agreement (±1.96 SD)] of the thyroid gland revealed that the majority of measured differences in densities between TNC and VNC images did not fall within the range of agreement (−/+ 15 HU) (Figure 2). In comparison, a Bland–Altman plot [95% limit of agreement (±1.96 SD)] of the sternocleidomastoid muscle revealed that the majority of measured differences in densities between TNC and VNC images fell within the range of agreement (−/+ 15 HU) (Figure 3).

SNR and CNR for all anatomical structures except for the carotid artery and jugular vein were significantly lower on VNC images compared to TNC images (Table 2 and Supplemental Table 3).

## Discussion

This study demonstrated that differences in densities between true non-contrast and virtual non-contrast images in the neck region were <15 HU for all anatomical structures, except the thyroid gland. The mean difference in density between TNC and VNC images of the thyroid was 53.2 HU (95% CI 46.8; 59.6, *p* = <0.001).

VNC images obtained through DECT acquisitions have been proposed as a means of reducing radiation exposure by replacing TNC images. The feasibility of VNC images as a substitute for TNC images has been investigated for conditions such as renal carcinoma,^21^ lung emphysema,^22^ and cancer affecting the upper aerodigestive tract.^23^ The results of this study revealed an acceptable agreement between VNC and TNC images with regards to the density of most anatomical structures in the neck. Therefore, VNC images may have the potential to serve as a substitute for TNC images in the neck. However, in this study, there was a significant discrepancy in the density of the thyroid between TNC and VNC images. These findings suggest that the feasibility of VNC images for thyroid imaging might be limited.

This discrepancy is explained by subtraction of the administered and intrinsic iodine of the thyroid on VNC images. Thyroid tissue has a higher intrinsic iodine content and thus a greater inherent density on TNC images, compared to surrounding normal and pathological structures such as parathyroid adenomas. The algorithm that generates VNC images subtracts the intrinsic iodine content of the thyroid, which may lead to reduced soft tissue contrast between the thyroid gland and surrounding tissue (Figure 1). Consequently, this may limit the utility of VNC images in evaluating thyroid and parathyroid pathology.

This finding is in concordance with three recent studies that have reported that VNC images were not useful for replacing TNC images in the detection of parathyroid adenomas using 4D-CT.^4,24,25^ Conversely, one study demonstrated that while the thyroid gland exhibited significantly lower density on VNC images, this did not appear to affect the diagnostic accuracy of parathyroid adenoma localization.^26^ This could be due to the fact that even on VNC images, the density of the thyroid gland remained considerably higher than that of parathyroid adenomas.^26^ A study investigating the utility of VNC images in detecting papillary thyroid carcinoma observed a significant decrease in mean density, image quality, and diagnostic accuracy of VNC compared to TNC images.^20^ As a result, the study concluded that VNC images cannot be directly used to replace TNC images in patients with papillary thyroid carcinoma.^20^ Further research is needed to evaluate the potential role of VNC images in the lower neck region.

While this study suggests that VNC images may have limited utility for evaluating the thyroid and surrounding tissue, VNC images may still have potential in evaluating other pathology in the neck region, such as salivary gland disease (sialolithiasis). For sialolithiasis, scanning protocols consisting of an unenhanced scan followed by a contrast-enhanced scan would result in increased radiation exposure.^8^ By employing DECT for the reconstruction of VNC images, it is possible to overcome this limitation. This approach eliminates the need for a two-phase scanning protocol, thereby reducing the additional radiation dose, while still enabling the identification of sialolithiasis. Hence, there is a need for future research that specifically focuses on VNC imaging for the detection of sialolithiasis.

Compared to TNC images, VNC images revealed a considerable reduction in CNR and SNR. This decrease in image quality could potentially make diagnosing neck pathology more challenging. These findings are consistent with a study conducted by Zhang et al,^18^ which also observed a similar decrease in CNR and SNR in neck VNC images. Nevertheless, the SNR/CNR values in VNC images may still meet diagnostic imaging standards.

This study has several limitations. Firstly, this was a single center study with a retrospective design. Furthermore, there are also limitations inherent to ROI placement, particularly given the small size of several lymph nodes. This was partly addressed by placing multiple ROIs in different LN’s in the same patient. This study did also not include multiple observers for ROI placement. Therefore, no data on the intra- or inter-observer agreement was available. Lastly, some scans were obscured by artefacts, making it difficult to accurately place the ROIs. Efforts were made to identify the most suitable location for each ROI.

In conclusion, virtual non-contrast imaging may be a viable alternative for the majority of structures in the neck region. However, due to significant differences in CT density of thyroid tissue, true non-contrast imaging cannot be directly substituted by virtual non-contrast imaging when examining the thyroid and its surrounding tissue. Further research is needed to determine the diagnostic utility of virtual non-contrast imaging in the neck region.

## Supplemental Material

Supplemental Material - Can virtual non-contrast imaging replace true non-contrast imaging in multiphase scanning of the neck region? Click here for additional data file. Supplemental Material for Can virtual non-contrast imaging replace true non-contrast imaging in multiphase scanning of the neck region? by Zaid Al-Difaie, Max HMC Scheepers, Nicole D Bouvy, Sanne Engelen, Bas Havekes and Alida A Postma in Acta Radiologica Open

## Ethical statement

### Ethical considerations

The study protocol was approved by the Medical Ethics Commission (17-4-077.2).

### Informed consent

Informed consent was waived.
