# Supplementary material for: Can virtual non-contrast imaging replace true non-contrast imaging in multiphase scanning of the neck region?
Source: Acta Radiol Open. 2023 Sep 25;12(8):20584601231205159. doi: 10.1177/20584601231205159 (PMC10521284; doi:10.1177/20584601231205159)
Supplement: Supplemental Material - Can virtual non-contrast imaging replace true non-contrast imaging in multiphase scanning of the neck region? [file sj-pdf-1-arr-10.1177_20584601231205159.pdf]

Online-Only Supplementary Material

Table 3. Differences in attenuation between true and virtual non contrast scans (50sec) for anatomical structures in the neck.

Tissue	TNC‡	VNC (50sec)‡	Difference (95% CI)	p-value
Thyroid gland	101.1 (25.1)	49.4 (15.7)	51.7 (45.8; 57.7)	< 0.001
Lymph node	51.8 (11.2)	37.2 (9.5)	14.6 (12.3; 16.9)	< 0.001
Carotid artery	50.7 (8.9)	44.2 (6.9)	6.5 (4.5; 8.5)	< 0.001
Jugular vein	49.3 (6.9)	43.0 (6.1)	6.2 (4.6; 7.8)	< 0.001
Sternocleidomastoid Muscle	60.9 (5.4)	52.5 (6.7)	8.4 (6.7; 10.0)	< 0.001
Fat	-112.7 (17.7)	-99.5 (11.7)	-13.2 (-16.7; -9.7)	< 0.001
SNR thyroid	9.2 (3.8)	4.5 (2.1)	4.7 (4.1; 5.3)	< 0.001
CNR thyroid	3.6 (2.6)	-0.3 (1.6)	3.9 (3.3; 4.6)	< 0.001
SNR Lymph node	4.8 (2.1)	3.4 (1.3)	1.3 (1.1; 1.6)	< 0.001
CNR lymph	-0.8 (1.2)	-1.5(1.3)	0.7 (0.4; 1.0)	< 0.001
SNR Carotid artery	4.7 (1.9)	4.0 (1.5)	0.6 (0.4; 0.8)	< 0.001
CNR Carotid artery	-0.9 (1.1)	-0.8 (0.8)	-0.1 (-0.4; 0.1)	.12
SNR Jugular vein	4.6 (1.8)	3.9 (1.4)	0.6 (0.5; 0.8)	< 0.001
CNR Jugular vein	-1.0 (0.8)	-0.9 (0.8)	-0.1 (-0.3; 0.1)	0.08

SNR Sternocleidomastoid				
Muscle	5.6 (2.0)	4.9 (1.8)	0.8 (0.6; 0.9)	< 0.001
SNR Fat	-10.6 (4.4)	-9.2 (3.5)	-1.3 (-1.7; -1.0)	< 0.001
CNR fat	-16.2 (6.3)	-14.1 (5.2)	-2.1 (-2.5; -1.6)	< 0.001

‡Data represent attenuation expressed in mean Hounsfield units with standard deviation between brackets.
CI: confidence interval, CNR; contrast to noise ratio, SD: standard deviation, SNR; signal to noise ratio, TNC true- non contrast, VNC; virtual non contrast,

Table 4. Differences in attenuation between arterial virtual non-contrast scan (30sec) and venous virtual non contrast scan (50sec) for anatomical structures in the neck.

Tissue	VNC (30sec)‡	VNC (50sec)‡	Difference (95% CI)	p-value
Thyroid gland	47.8 (16.2)	49.4 (15.7)	-1.7 (-4.2; 0.9)	0.194
Lymph node	37.7 (8.4)	37.2 (9.5)	0.5 (-1.4; 2.5)	0.602
Carotid artery	43.4 (7.3)	44.2 (6.9)	-0.8 (-2.6; 1.1)	0.410
Jugular vein	42.8 (6.6)	43.0 (6.1)	-0.3 (-1.8; 1.2)	0.728
Sternocleidomastoid Muscle	52.8 (6.0)	52.5 (6.7)	0.3 (-1.1; 1.7)	0.687
Fat	-100 (11.1)	-99.5 (11.7)	-0.5 (-2.0; 1.0)	0.486

‡Data represent attenuation expressed in mean Hounsfield units with standard deviation between brackets.
CI: confidence interval, SD: standard deviation, TNC true- non contrast, VNC; virtual non contrast.
